# Biophysical and Biochemical Assays for Screening Small Molecule Inhibitors Targeting Toxin–Ribosome Interactions

**DOI:** 10.3390/toxins18060267

**Published:** 2026-06-16

**Authors:** Eric J. Bryan, Vishal Vijayanand, Xiao-Ping Li, John E. McLaughlin, Michael Pierce, Arkajyoti Dutta, Nilgun E. Tumer

**Affiliations:** 1 Department of Plant Biology, School of Environmental and Biological Sciences, Rutgers, The State University of New Jersey, 59 Dudley Road, New Brunswick, NJ 08901, USA; ejb236@dls.rutgers.edu (E.J.B.); xpli@sebs.rutgers.edu (X.-P.L.); mclaughj@sebs.rutgers.edu (J.E.M.); mdpierce@sebs.rutgers.edu (M.P.); 2 Department of Bio-Sciences, School of Bio Sciences and Technology, Vellore Institute of Technology, Vellore 632014, Tamil Nadu, India

**Keywords:** infectious disease, ricin toxin, Shiga toxin, drug screening, drug discovery

## Abstract

Ribosome-inactivating proteins are a class of toxins that target eukaryotic ribosomes, inhibit protein synthesis, and ultimately induce cell death. Several of these toxins pose significant clinical and public health threats. Among these, ricin, derived from the castor bean plant (*Ricinus communis*), is a highly potent biotoxin with recognized bioterrorism potential. Other ribosome-inactivating proteins, including Shiga toxin produced by pathogenic *Shigella* and *Escherichia coli*, as well as mucoricin from Mucorales fungi, contribute to disease severity and can lead to life-threatening complications. Despite these risks, no approved therapeutics are currently available. The development of effective inhibitors depends on robust and well-defined strategies to identify and validate small molecules that disrupt toxin–ribosome interactions. Efforts to target the catalytic active site have met with limited success, largely due to its broad, shallow, and highly polar architecture, which is not conducive to high-affinity binding by drug-like molecules. In contrast, the ribosome-binding interface represents a more tractable target, as it is essential for toxin recruitment and offers more structurally defined and druggable features. Inhibitors targeting this interface can also exert allosteric effects by disrupting long-range conformational coupling between the ribosome-binding region and the active site, thereby attenuating catalytic activity without directly engaging the catalytic pocket. In this review, we compile and evaluate biophysical and biochemical assays for the discovery and characterization of small-molecule inhibitors that target toxin–ribosome interactions. We examine in vitro binding approaches, including surface plasmon resonance-based fragment screening and fluorescence anisotropy assays for ranking inhibitory activity. We further review biochemical and molecular assays that assess ribosome protection from toxin-mediated depurination, along with complementary cell-based assays that evaluate functional rescue in cellular systems. Collectively, this review consolidates current screening methodologies and highlights opportunities to refine assay strategies, thereby supporting the advancement of targeted therapeutics.

## 1. Introduction

Ribosome-inactivating proteins (RIPs) are toxins produced by a wide variety of organisms across the kingdoms of life, including bacteria, fungi, and plants [1]. They bind to and inactivate the eukaryotic ribosome, stopping protein synthesis and inducing cell death [2]. RIPs are classically categorized into two distinct types: type 1 and type 2 [3]. Type 1 RIPs are the simplest in structure, consisting of only a single catalytic domain with *N*-glycosidase activity [1]. Type 2 RIPs are heterodimers that contain two distinct domains, a catalytically active A chain and a cell-binding B chain, which mediates cellular entry of the toxin [1]. The AB domains are fused by a disulfide bond that is intracellularly reduced, releasing the catalytic A chain [4]. A rare third type of RIP (type 3) has been described, where the catalytic A chain is fused to a B chain as a precursor that must be cleaved for activity [5,6].

The environmental and biomedical importance of RIPs is extensive. Type 1 RIPs are primarily plant antiviral proteins and include pokeweed antiviral protein, trichosanthin (TCS), and mirabilis antiviral protein (MAP30) [1]. Type 1 RIPs are generally inactive in humans due to poor cellular uptake [7]. In contrast, many type 2 RIPs have been identified as highly toxic to humans [7]. Both ricin, produced by the castor bean plant, and abrin, produced by the rosary pea plant, have lethal doses in humans as low as 5 µg/kg and 0.1 µg/kg, respectively [8,9]. Both toxins are easily extracted from their respective plants and can be formulated into weaponizable agents, making them significant concerns for bioterrorism [10]. Indeed, several high-profile incidents have been reported over the past several decades. This includes a high-profile assassination of a Romanian journalist in the 1970s and attempted assassinations of two U.S. presidents with letters that contained ricin powder [11,12,13]. Shiga toxin (Stx), produced by pathogenic strains of *Shigella* and *Escherichia coli*, and mucoricin, produced by Mucorales fungi, cause debilitating infections in humans [14]. Shiga-associated bacterial infections, which total over 2.8 million annual cases, can cause bloody diarrhea and a dangerous kidney condition called hemolytic uremic syndrome [15,16]. Mucoricin is a recently discovered and critical pathogenicity factor for mucormycosis infections by various molds known as *Mucormycetes*. The common causative agents include *Rhizopus* species, *Mucor* species, *Lichtheimia* species, *Apophysomyces*, and *Cunninghamella bertholletiae*. These fungi are common in the environment and are generally acquired by inhaling spores or through skin inoculation through burns, cuts, or wounds [17]. Wound-acquired infections are especially prevalent in immunocompromised individuals [17]. The overall mortality rate of mucormycosis infections is approximately 50% [17].

The B domain of type 2 RIPs is a cell-binding domain required for cellular entry. The B subunits of ricin or Shiga toxin closely mimic a lectin and bind to carbohydrates (i.e., galactose for ricin or the trisaccharide component of Gb3 for Stx) on the cell surface [18,19,20]. The toxin-carbohydrate complexes are then internalized and undergo retrograde transport to the Golgi and, subsequently, the endoplasmic reticulum [21,22,23]. The disulfide bond linking the A and B chains is reduced within the ER, and the A chain then coopts the endoplasmic reticulum-associated degradation system for localization to the cytosol, wherein it exerts its depurination activity on ribosomes [4,24].

The primary function of the A subunit of RIPs is the removal of a highly conserved adenine residue (A_4324_ in rat rRNA) from the sarcin–ricin loop in the 28S rRNA of the large 60S eukaryotic ribosomal subunit via the toxin’s *N*-glycosidase activity [25]. The sarcin–ricin loop is a critical interaction site for translational GTPases, such as eukaryotic elongation factor 2, and plays a crucial role in the hydrolysis of GTP by these factors [26]. Depurination of the sarcin–ricin loop prevents the interactions with the translational GTPases and inactivates the ribosome, halting protein synthesis [26]. Unlike RIPs, ribotoxins such as α-sarcin inactivate the ribosome through a distinct RNA hydrolase activity wherein the toxin directly cleaves the 28S rRNA (between G_4325_ and A_4326_ in rats) and prevents sarcin–ricin loop function [27,28]. Depurination or endonucleolytic cleavage of the sarcin–ricin loop is irreversible and permanently disables the ribosome [26]. Accumulation of depurinated ribosomes induces the ribotoxic stress response and leads to the eventual death of intoxicated cells [7] by inducing apoptosis, underscoring their multifaceted role in mediating cell death [29,30,31].

To exert depurination activity, the catalytic subunit of RIPs interacts with the eukaryotic ribosome by binding to the ribosomal P-stalk proteins [32,33]. The eukaryotic P-stalk is a pentamer, which consists of two P1-P2 heterodimers that are attached to the P0 protein [34,35]. The C-termini of the stalk proteins contain an identical sequence. TCS and the A subunits of ricin (RTA), Shiga toxin (StxA1), and maize RIP bind to the C-terminus of the ribosomal P-stalk via an 11-amino acid conserved sequence, SDDDMGFGLFD [32,33,36,37,38,39,40,41]. These interactions occur in a distinct toxin P-stalk binding pocket distal from the catalytic cleft and depend on hydrophobic and electrostatic interactions between the P-stalk and the toxin [38,40,42,43]. Mutational studies have demonstrated that this interaction is critical for efficient toxin depurination. Loss of the ribosomal P-stalk decreases toxin activity by 200-fold [44]. Therefore, the association of RIPs with P-stalk proteins is an essential step for RIP function.

Currently, there are no U.S. Food and Drug Administration-approved therapeutic interventions for infections or intoxications associated with RIPs. To address this critical gap, multiple research groups have initiated efforts to develop effective therapeutic strategies. Early studies primarily focused on the development of inhibitors targeting the catalytic site of RIPs by structure-based design [45,46,47,48,49,50]. Transition state analogs [51] and “doorstop inhibitors” were designed to target the catalytic site of RIPs and directly block the depurination of the eukaryotic ribosome [52]. However, none of the doorstop inhibitors showed significant activity in cell-based assays (<25% cell protection at 30 µM inhibitor) or in vivo [52]. Several compounds identified in Vero cell protection assays exhibited only weak inhibition of RTA catalytic activity in cell-free systems, suggesting that their protective effects were not mediated through direct inhibition of the toxin’s active site. Instead, these compounds likely acted as cell death inhibitors that mitigated ricin-induced cytotoxicity through downstream cellular mechanisms. For example, CID 16271106, identified by Wahome and colleagues, protected Vero cells from ricin intoxication with an *EC*_50_ of approximately 30 μM, yet inhibited RTA-mediated protein synthesis in a cell-free translation assay only at concentrations approaching 1 mM. This marked disparity in potency indicates that the compound’s cellular protective effect was unlikely to result from direct inhibition of RTA enzymatic activity and was more consistent with modulation of toxin-induced cell death pathways [53].

Although the active site of ricin (and related RIPs such as Shiga toxin) is relatively large, it is highly polar and adapted to bind ribosomal RNA, making it a poor match for typical drug-like molecules. Compounds that can engage it effectively tend to be charged, bulky, and poorly cell-permeable, while smaller, drug-like molecules bind too weakly to compete. Even when binding occurs in vitro, RIPs are extremely efficient enzymes, so inhibition must be essentially complete, and the inhibitor must be present in the cytosol when the toxin arrives. Most compounds fail to achieve inhibition due to cellular delivery barriers and timing issues. In addition, the toxin operates in the context of abundant ribosomes and auxiliary binding interactions, further reducing the effectiveness of competitive inhibitors. Many active-site mimics also risk off-target toxicity because they resemble nucleic acid substrates. Together, these factors explain why no active-site inhibitors translate into robust activity in cell-based assays.

Additional approaches have explored inhibitors that disrupt the retrograde trafficking of RIPs to the endoplasmic reticulum following cellular entry. One notable example is Retro-2, which has been shown to protect mice from lethal doses of ricin. However, its precise cellular target remains unknown [54,55,56]. In parallel, vaccine development against ricin has advanced with the recombinant candidate RiVax, currently under development by Soligenix. RiVax has demonstrated protective efficacy against ricin exposure and is undergoing clinical trials in the United States [57]. Despite these promising results, vaccine-based approaches do not address acute toxin exposure or infections caused by RIP-producing pathogens.

Several reviews have examined individual methodologies used in RIP research, providing valuable insights into specific aspects of toxin characterization and inhibitor evaluation. For example, Zhou et al. comprehensively reviewed functional assays used to measure RIP catalytic activity, highlighting approaches for assessing toxin function and inhibition [58]. Similar reviews primarily focus on individual assay platforms and do not address how these methods can be integrated into a systematic inhibitor discovery pipeline. Likewise, strategies for hit identification, binding analysis, and cellular validation have generally been discussed separately in the literature, resulting in a fragmented view of the overall drug discovery process.

To our knowledge, no previous review has consolidated the sequential experimental steps required for the discovery and characterization of RIP inhibitors into a unified framework. The novelty of the present review lies in its integration of both in vitro and cell-based methodologies into a stage-wise workflow that guides the progression of candidate compounds from initial hit identification through mechanistic validation and biological evaluation. By linking complementary screening, binding, functional, and cellular assays, this review provides a practical roadmap for prioritizing and advancing RIP inhibitor candidates. Collectively, the approaches discussed herein establish a cohesive strategy for the discovery and characterization of small-molecule inhibitors targeting toxin–ribosome interactions.

Our drug discovery efforts have targeted ricin and Shiga toxin 2 (Stx2) directly by blocking the essential interaction between the A subunits of these RIPs (RTA for ricin, and Stx2A1 for Stx2) and the ribosomal P-stalk [59,60,61,62,63]. In this review, we describe the experimental methodologies used to develop small-molecule inhibitors targeting RIPs to facilitate efficient drug discovery. We examine several assays designed to directly measure the binding affinity of inhibitor candidates to RIP toxins, including surface plasmon resonance (SPR) and fluorescence anisotropy (FA)–based competition assays. We also introduce additional experimental approaches that can strengthen current inhibitor discovery pipelines, including Förster resonance energy transfer (FRET)–based assays. In addition, we describe complementary orthogonal assays that validate binding candidates through direct functional readouts. We focus on a widely used quantitative reverse transcription PCR (qRT-PCR) assay developed in our laboratory, capable of measuring rRNA depurination levels in a microplate format, enabling rapid, efficient assessment of inhibitor candidates for protective activity following toxin exposure. We further discuss cell-free translation assays that assess protein synthesis inhibition. Both approaches can be applied in the presence of candidate inhibitors to evaluate their ability to protect against ribosome depurination. Finally, we present the last assay at the cellular level to test the final effect of the inhibitor’s function, the cellular viability assay, which quantifies candidate inhibitors efficacy at protecting cells from RIP-induced death. Collectively, the methodologies outlined in this review aim to support the development of RIP-targeted therapeutics capable of treating life-threatening toxin exposures and infections, while also contributing to national preparedness against potential bioterrorism threats.

## 2. Surface Plasmon Resonance (SPR) for Fragment Screening and Determination of the Binding Affinity of the Fragments

SPR-based biosensing instruments originated from laboratory demonstration designs developed for instructional use in the early 1980s [64]. The first commercially available SPR instrument for measuring biomolecular interactions, BIAcore, was introduced in 1990 by Pharmacia Biosensor [65]. Since then, multiple generations of SPR platforms have been developed, alongside a variety of related instruments introduced by other manufacturers under different proprietary names. SPR-based biosensing has become a widely adopted technique in both academic research and the pharmaceutical industry, particularly for studying biomolecular interactions and supporting drug discovery efforts. In this review, we provide a brief overview of the application of SPR, specifically using Biacore systems to characterize interactions of ricin and Shiga toxins with the ribosome, ribosomal P-stalk proteins, P-stalk peptides, and small molecules. We further highlight the utility of SPR in fragment-based screening approaches to identify initial hit compounds for inhibitor development against ricin and Shiga toxins, particularly in contexts where alternative techniques may be less effective.

Over the past 15 years, Biacore-based methods have been extensively developed to investigate interactions between toxins and ribosomes, protein complexes, peptides, small molecules, and fragment libraries. In most applications, untagged or N-terminal 10×His-tagged toxins are either covalently immobilized or captured on the sensor chip to generate an active surface. For covalent immobilization, amine coupling to CM5 sensor chips is most commonly employed. Reference surfaces are prepared either by activating and deactivating an unmodified surface or by immobilizing a non-interacting control protein to compensate for bulk refractive index and charge effects. In capture-based assays, N-terminal 10×His-tagged RTA or StxA1 is captured on an NTA sensor chip, and a similarly tagged, non-binding protein is captured on the reference surface to serve as a negative control to account for nonspecific interactions with the chip matrix in addition to bulk refractive index. Interaction partners are injected over both active and reference surfaces under near-physiological conditions. Binding responses are calculated by subtracting the reference signal from that of the active surface, enabling assessment of binding specificity. SPR measurements further provide quantitative kinetic and equilibrium parameters, including association and dissociation rate constants and binding affinities. These data are critical for target validation, initial hit identification, and subsequently lead optimization in inhibitor development.

We investigated the interactions of RTA and Stx2A1 with ribosomes and ribosomal P-protein stalk complexes purified from genetically modified yeast strains [32,39,66,67,68,69]. We identified the ribosome-binding sites of RTA and Stx2A1 by measuring the binding of ribosomes isolated from the mutant yeast strains and peptide mimics corresponding to the conserved C-terminal region of the P-stalk proteins. Site-directed mutagenesis was used to identify critical residues on RTA and Stx2A1 [33,44,70]. We showed that P-stalk binding sites are located distal to the catalytic active centers on the opposite face of each protein [42,43,71]. The peptide mimics demonstrated inhibitory activity, with *IC*_50_ values in the low micromolar range, comparable to their binding affinities, indicating competitive inhibition of toxin–ribosome interactions [72,73]. These findings established that the ribosome-binding interface represents a viable and previously unexplored target for inhibitor development. Structural analyses further validated these results. X-ray crystal structures of RTA and Stx2A1 in complex with the peptide mimics [37,73,74], together with X-ray and cryo-EM structures of the Stx2 holotoxin bound to P11 peptide [40] and the P-stalk pentamer [38], confirmed the identified binding sites and provided insights into the molecular interactions. These data enabled the rational design of inhibitors targeting ribosome binding.

Fragment-based drug discovery has been widely and successfully applied in the development of anticancer therapeutics [75]. This approach utilizes libraries of low-molecular-weight fragments (typically <300 Da) with diverse chemical scaffolds, enabling efficient identification of initial binding hits and characterization of key interactions within the target site. These fragments can subsequently be optimized through fragment growing or linking strategies to generate more potent lead compounds. Although fragment hits typically exhibit weak affinities in the millimolar range and limited biological activity, fragment-based drug discovery offers high hit rates and requires screening of relatively small libraries, usually consisting of a few thousand compounds. However, it necessitates highly sensitive detection methods and well-controlled experimental systems.

Because RIPs directly target ribosomes, their expression, particularly that of Stx2A1, remains challenging in vitro. In this context, SPR-based approaches provide a powerful platform, combining high sensitivity with low sample consumption. SPR is therefore particularly well suited for fragment library screening for toxins and for detecting weak fragment–protein interactions essential to fragment-based drug discovery efforts. Fragment-based screening by SPR generally involves three sequential steps.

**Figure 1:**
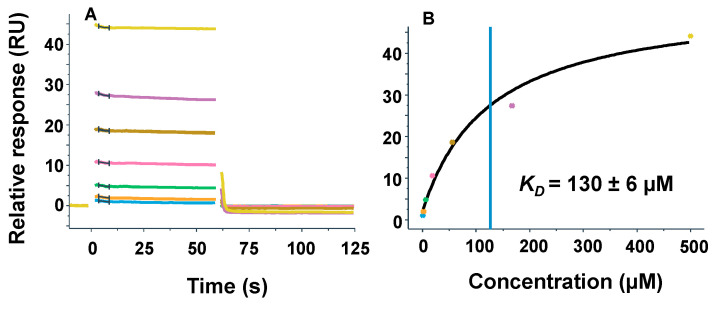
The interaction sensorgrams (**A**) and steady-state fitting (**B**) of a fragment with RTA. Biacore 8K^+^ was used to measure the interaction and determine the binding affinity (*K_D_*). RTA was immobilized on the active flow cell of a CM5 chip up to 5800–6000 response units using amine coupling. The reference flow cell was activated and blocked as reference. Fragment at concentrations of 0.7, 2.1, 6.2, 18.5, 55.6, 166.7 and 500 µM was passed over both surfaces at 30 µL/min for 60 seconds and dissociation was for another 60 seconds. The running buffer was PBS-P (20 mM phosphate, 2.7 mM KCl, 137 mM NaCl, and 0.05% surfactant P20, pH 7.4) with 2% DMSO. Any buffer mismatch was subtracted from the reference cell (Fc1) and solvent corrected. Fragment was run on 4 different channels as replicates. Data were analyzed using Biacore Insight Evaluation Software v. 5.0. The colored lines in panel (**A**) show the different concentrations of fragments, and the color dots in panel (**B**) show the binding levels, which match the colors used for the concentrations in panel (**A**). The vertical blue line indicates that the highest concentration used for the measurement is high enough to give a reliable *K_D_*.

**(1)**  Clean screen. In this initial step, the fragment library is screened against the empty surface of the selected sensor chip. Fragments that exhibit non-specific binding to the chip matrix are excluded from further analysis.
**(2)**  Binding-level screen. The target toxin is immobilized on the active surface of the sensor chip at an appropriate level, which is determined by the molecular weights of both the toxin and the fragments. For RTA and Stx2A1, immobilization levels typically exceed 5000 response units. Covalent immobilization on CM5 chips is commonly employed as a first approach, as it provides stable attachment and random ligand orientation, an important consideration when the binding site is unknown. Phosphate-buffered saline with surfactant (PBS-P) is frequently used as the running buffer. Fragment libraries are typically supplied in 96-well plates at 100 mM in 100% DMSO; therefore, careful matching of DMSO concentrations between running and sample buffers is essential, and DMSO correction is routinely applied during analysis. Fragments are injected at a single concentration, typically 50–200 µM. Fragment–protein interactions are characterized by rapid association and dissociation kinetics, resulting in square-shaped sensorgrams (Figure 1A). Short injection cycles (e.g., 30 s association and 30 s dissociation) are generally sufficient, and regeneration is often unnecessary. Modern Biacore SPR instruments enable screening of more than one thousand fragments within 24 h under these conditions. Positive and negative controls are periodically injected throughout the experiment to monitor surface integrity and baseline stability. Data from the single-concentration screen are processed using evaluation software, including DMSO correction, reference subtraction, and molecular weight normalization. Fragments displaying binding responses exceeding theoretical maxima or exhibiting non-ideal kinetics (e.g., slow association or slow dissociation) are excluded. The response threshold for hit selection is determined empirically.
**(3)**  Binding affinity determination. Selected fragments are advanced to dose–response analysis, typically using a series of five concentrations. Binding affinities are estimated by fitting the steady-state responses (Figure 1B), as the rapid kinetics of fragment interactions preclude determination of association and dissociation rate constants.

While SPR effectively identifies specific fragment–target interactions, it does not directly provide information about binding sites, and fragments may bind to multiple regions on the protein. Therefore, initial hits require further validation. Comparative binding studies using wild-type toxins and site-directed mutagenesis can help confirm target engagement. Orthogonal techniques, such as fluorescence polarization/anisotropy assays, are needed for additional validation. In most cases, high-resolution structural methods, such as X-ray crystallography, are required to definitively identify binding sites and to provide detailed interaction information necessary for hit optimization. However, the inherently low affinities of fragment hits can pose challenges for both biochemical and structural characterization.

During the hit-to-lead and lead-optimization stages, optimized fragments and lead compounds are routinely characterized by SPR to determine their binding kinetics and affinities. In these experiments, a series of at least five concentrations, typically including a repeat injection of one concentration at the end of the series for quality control, is injected over the immobilized target surface. Binding affinities can be derived either by kinetic fitting of the sensorgrams or by steady-state analysis, depending on the interaction profile.

For lead compounds exhibiting slower dissociation rates, which are often indicative of higher-affinity interactions, it is necessary to establish suitable regeneration conditions to fully restore the sensor surface between injections. This step is critical to ensure accurate and reproducible affinity measurements. At this stage, SPR provides key parameters, including association and dissociation rate constants as well as binding specificity, all of which are essential for guiding rational compound optimization. Using Fragment-based drug discovery with Biacore SPR instruments, hits targeting the ribosome-binding sites of RTA and Shiga toxin 2 have been successfully identified [60,63]. Subsequent optimization of RTA-targeting hits has yielded lead compounds with low micromolar binding affinities and measurable inhibitory activity against ricin holotoxin in cell-based assays [59,61,62]. However, despite these improvements, the lead compounds continue to exhibit rapid association and dissociation kinetics. Further optimization is therefore required, particularly to reduce dissociation rates, in order to enhance overall binding affinity and improve therapeutic potential.

## 3. Fluorescence Anisotropy (FA) Assays to Measure Interactions of RIPs with P-Stalk Peptides and to Rank Small Molecules That Inhibit Toxin Binding

FA assays are homogeneous analytical techniques that measure the anisotropy emitted by a fluorescent molecule. When a fluorophore is excited by plane-polarized light, the fluorescence emitted depends upon the rotational mobility of the molecule during its excited lifetime [76,77,78]. The in vitro binding assays utilize this property to differentiate between the free and bound fluorescent probe. Free fluorophores rotate rapidly in the solution, producing a low anisotropy signal. In contrast, bound probes rotate slowly and produce higher anisotropy signals. In a competition-based experimental model, compounds or peptides competitively displace the bound probe, restoring its rotational mobility and reducing anisotropy.

An FA-based competition assay has been developed to study the specific interactions of RIPs with the P-stalk peptide. A fluorescently labeled P11 peptide was generated that mimics the C-terminal domain sequence of the P-stalk. The fluorescent probe (BODIPY-TMR-X N-hydroxysuccinimide) is attached to the N-terminus of the P11 peptide [79]. The P11 probe was first competed with unlabeled competitor peptides truncated from P11 to P3 and was used to identify the residues, which displayed strong binding affinity to the toxin subunit. Unlabeled P11 peptide displayed greater binding compared to other truncated peptides (P10-P3) [79]. The N-terminal DDD residues were found to be essential, as they drastically reduced binding to RTA and displayed moderate binding to Stx2A1 [79]. In contrast, any loss in the GFGLFD motif led to the absence of binding to either RIP [79].

Results with the unlabeled peptides and early-stage binding inhibitors confirmed that the FA assay can measure site-specific affinities more accurately than SPR. Notably, some acidic peptides appeared to bind the toxins in SPR but failed to displace the fluorescent probe in the FA assay, suggesting that these interactions were driven by non-specific electrostatic effects rather than true binding [79]. In addition, when suboptimal analyte concentrations are used, SPR may not accurately determine the affinities of small molecules that produce minimal refractive index changes upon binding, limiting the extraction of association and dissociation rate constants [60,80,81,82]. The FA assay detects binding in solution by measuring changes in rotational mobility, enabling precise affinity measurements even for weakly interacting small molecules. Importantly, FA-based screening methods to rank inhibitor candidate binding affinity are commonplace in early hit identification and optimization strategies and have been utilized in the development of several preclinical and clinical drugs, including the BCL-2 inhibitors ABT-263 (Navitoclax, AbbVie) and Venetoclax (AbbVie) [83,84]. Overall, these findings demonstrate that a FA-based competition assay is robust, quantitative, and well-suited for high-throughput screening (HTS) or ranking of peptide analogs or small molecules that inhibit toxin–ribosome interactions with the P-stalk, providing valuable insights into the development of therapeutics targeting RIPs.

### 3.1. Quantitative FA Binding Analysis—P-Stalk Probe Binding Parameters

FA assays allow quantitative measurement of binding strength and competitive inhibition without the requirement of any separation or washing steps [85]. A comprehensive guide for the quantitative analysis of the FA competition assay is described below.

**Figure 2:**
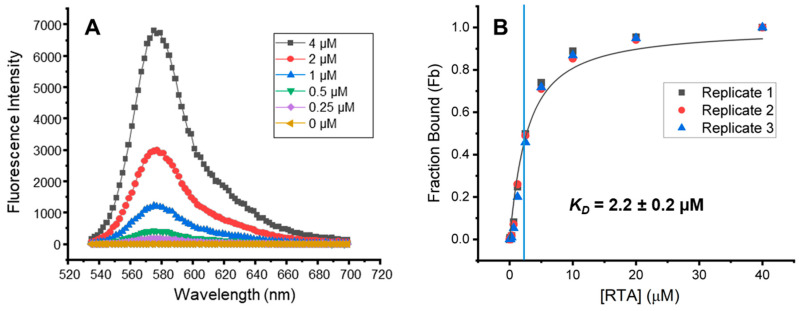
Fluorescence emission scans of the BODIPY-TMR-X-P11 fluorescent probe (**A**) and probe binding curve with RTA (**B**). (**A**) Varying concentrations of the fluorescent probe were prepared in FA buffer (25 mM Tris-HCl pH 8.0, 100 mM NaCl) and scanned using a BioTek Synergy 4 fluorescent plate reader (Agilent, Santa Clara, United States) with the excitation wavelength fixed at 495 nm. (**B**) Increasing concentrations of protein were combined with probe in 96-well plates (Corning #3993). The final volume in each well was 40 µL, and the final concentrations were 0–40 µM RTA and 1 µM probe. The plate was scanned using the BioTek Synergy 4 equipped with excitation and emission filters of 530/25 nm and 590/35 nm, respectively, to generate anisotropy values. The fraction bound at each protein concentration was calculated using Equation (1), and then the resulting binding curve was generated by plotting the fraction bound values versus protein concentration with Equation (2) using Origin 2024(b). Three independent replicates are shown, and the resulting *K_D_* and standard error is indicated by the blue line.

First, the optimal concentration of the fluorescent ligand (probe) for the assay must be determined. This can be accomplished by measuring the emission spectra of a range of probe concentrations with a fluorimeter (Figure 2A) [79]. The fluorimeter used should be the instrument utilized for the subsequent FA competition assay. The concentration that gives an ~20-fold higher signal than the buffer background is optimal for the competition assay, as it conserves reagents while minimizing the background emission contribution to the experiment (Figure 2A) [79]. It is important that the buffer and temperature chosen for these initial experiments be maintained consistently throughout all subsequent experiments. Small changes in buffer constituents or experimental temperature can have a large effect on FA results, compromising HTS comparisons.

Next, the binding parameters for the probe and protein need to be established. Fraction bound (*F_b_*) represents the proportion of probe that is bound to the target protein under equilibrium conditions [79,86]. In practice, not every probe molecule will occupy the binding site, since binding depends on both ligand concentration and the affinity of the interaction. Establishing an optimal fraction bound is critical for competition assays, as too little probe binding results in a weak FA signal, whereas complete saturation prevents displacement by competing with the inhibitors [87,88]. Typically, assays are designed so that the *F_b_* is between 50–70%, maximizing signal strength while allowing inhibitors to compete effectively at relatively low concentrations [79,86]. This increases the sensitivity of the assay to detect weak to moderate binders [79,86]. The fraction bound is related to the experimental anisotropy values using Equation (1):

(1) $$F_{b}=\frac{A_{obs}-\;A_{free}}{A_{bound}-A_{free}}$$

where *A_obs_* is the observed anisotropy at a given protein concentration, *A_free_* is the anisotropy of an unbound/free probe, and *A_bound_* is the anisotropy of the bound probe.

Subsequent FA competition assays maintain the *F_b_* value, corresponding to a specific protein concentration, while keeping the ligand (fluorescent probe) concentration fixed. The equilibrium dissociation constant (*K_D_*), defined as the binding affinity between the protein and the fluorescent probe, can be obtained by varying protein concentration in a dose-dependent manner and calculating the *F_b_* at each dose using Equation (1) [88,89]. The *A_free_* is the anisotropy of a control containing only the probe, with no protein. The *A_bound_* is the anisotropy at the highest tested protein concentration, which is at or near saturation. A low *K_D_* indicates tight binding, whereas a high *K_D_* indicates weaker binding [78,88]. Probe ligands with *K_D_* ~ 0.1 µM are often preferred, as they provide strong anisotropy signals and stable binding [86,90]. The *K_D_* can be obtained from substituting the *F_b_* value in Equation (2):

(2) $$F_{b}=\frac{K_{D}+Lt+Rt-\sqrt{\left\{{{(K}_{D}+Lt+Rt)}^{2}-4LtRt\right\}}}{2Lt}$$

where *Lt* is the concentration of the probe, and *Rt* is the concentration of the protein at a specific *F_b_*. The *F_b_* is chosen to be between 0.5–0.7, and the *K_D_* is determined from the equation.

Graphically, when *F_b_* is plotted as a function of protein concentration, the binding relationship forms a sigmoidal curve. The base of the curve denotes free probe in solution (*A_free_*), and the top of the curve is when the probe concentration is highly saturated with protein (*A_bound_*). In practice, the *F_b_* and *K_D_* can be simultaneously determined from the dose–response curve using graphing software (Figure 2B).

In summary, the probe binding experiments establish all the required parameters needed for the FA competition assay, namely:

1. The optimal probe concentration (*Lt*);
2. Fraction bound (*F_b_*);
3. The protein concentration (*Rt*) corresponding to that specific *F_b_*;
4. The *K_D_* of the probe for the protein.

### 3.2. Quantitative Binding Analysis—Screening Small Molecule Compounds for P-Stalk-Specific Binding

After the probe’s binding parameters are established, screening small molecules targeting the toxin P-stalk binding site can be performed. Inclusion of small molecule inhibitors that displace the fluorescently labeled ligand already bound to the protein decreases the anisotropy signal. Thus, an increase in the concentration of the inhibitor molecules leads to higher displacement of the labeled probe and a reduction in the anisotropy signal [79]. In the FA competition model, a dose–response curve is generated by varying the inhibitor concentration and keeping the experimental conditions defined in Section 3.1 fixed. The percent binding of inhibitor to protein is calculated using Equation (3):

(3) $$Inhibitor\;Bound\;\left(\%\right)=\;\frac{Abound-Aobs}{\left(Abound-Aobs\right)Q+(Aobs-Afree)}\;\times \;100$$

where *A_obs_* is the observed anisotropy at a given inhibitor concentration, *A_bound_* is the anisotropy of the fully bound probe, *A_free_* is the anisotropy of the free probe, and Q is the quantum yield.

As in Section 3.1, *A_free_* is calculated from the probe-only control. A_bound_ is calculated from a control that contains the protein and probe, but no inhibitor. Quantum yield (*Q*) is defined as the ratio between the number of photons emitted and the number of photons absorbed [76,78]. A *Q* value close to 1 indicates the successful emission of a photon as fluorescence, whereas a *Q* value of 0 indicates that the excitation energy has been lost through non-radiative processes such as heat emission [86,91]. Dyes that provide high quantum yield, such as BODIPY FL and TMR series, are usually preferred for a strong fluorescence signal, as they maintain the excited emission state for a longer period of time [87,92]. In small molecule screening, where probe displacement induces minute anisotropy shifts, a bright, high quantum yield fluorophore with a strong fluorescence signal leads to better detection, improved assay sensitivity, and reduced background noise [87].

**Figure 3:**
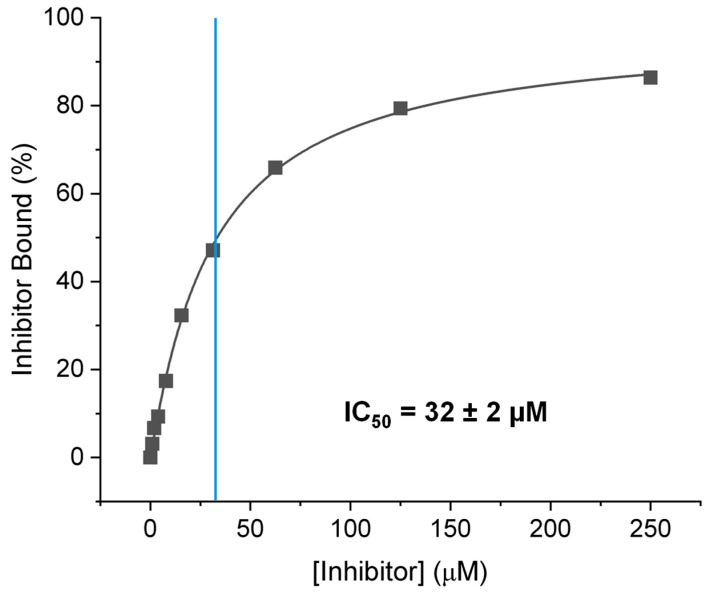
Competition binding curve of a P-stalk binding inhibitor to RTA. Reaction mixtures were prepared in a 96-well plate (Corning #3993). Each well contained 0–250 µM inhibitor, 4.5 µM RTA, and 1 µM probe prepared in FA buffer, with the final volume in each well was 40 µL. The plate was centrifuged at 400× *g* for 2 minutes, then incubated in the dark for 30 minutes. The plate was then scanned using a BioTek Synergy 4 plate reader equipped with excitation and emission filters of 530/25 nm and 590/35 nm, respectively, to generate anisotropy values. The resulting percent inhibitor bound was calculated for each inhibitor concentration using Equation (3), and then the percent inhibitor bound was plotted versus inhibitor concentration with Equation (4) using Origin 2024(b). The percent bound at each inhibitor concentration are indicated by the points, and the black line represents the competition binding curve generated by Equation (4). The *IC*_50_ value of the competition curve is indicated by the blue line.

Graphically, the percent inhibitor bound at each inhibitor concentration also forms a sigmoidal curve, which in turn displays the relationship between inhibitor concentration and the amount of inhibitor bound to the protein (Figure 3). The percent inhibitor bound is inverse to the percent probe bound, as increased inhibitor binding displaces the probe from the protein. The base of the curve represents where probe concentration is highly saturated, and no inhibitor is bound, whereas the top of the curve denotes free probe at a highly saturated inhibitor concentration (Figure 3).

The *IC*_50_ is the concentration of inhibitor required to reduce probe binding by 50% [86]. It is determined from the competition binding curve, which is generated by plotting percent inhibitor bound versus inhibitor concentration with Equation (4): [86]

(4) $$Inhibitor\;Bound\;(\%)=V_{max}\frac{{\left[C\right]}^{nH}}{{\left({IC}_{50}\right)}^{nH}+{\left(\left[C\right]\right)}^{nH}}$$

where V_max_ is the maximal binding percentage, [C] is inhibitor concentration, *IC*_50_ is half maximal inhibitory concentration, and nH is the Hill coefficient, which is a measure of cooperativity between the inhibitor and protein.

*IC*_50_ values are highly assay-dependent because they are influenced by probe concentration, probe affinity, and assay conditions such as temperature and ionic strength [91]. Thus, while *IC*_50_ is useful for ranking compounds within the same experiment, conversion to *Ki* is required for cross-experiment or cross-laboratory comparisons. *K_i_* is the inhibitory binding constant that reflects the affinity of a competing inhibitor for the same binding site occupied by the fluorescent probe. *Ki* is obtained by substituting the experimental *IC*_50_ value and probe binding parameters into Equation (5):

(5) $$K_{i}=\;\left[\frac{IC50}{\left\{\frac{F0\;K_{D}}{\left(1-F0\right)\left(2-F0\right)}\right\}+\left\{\frac{F0\;Lt}{2}\right\}}-1\right]\left\{\frac{K_{D}\;F0}{2-F0}\right\}$$

where *Lt* is the concentration of the fluorescent probe, *K_D_* is the probe dissociation constant, *F*0 is the fraction bound, and *IC*_50_ is the concentration of the inhibitor at which 50% inhibition is observed.

The *Ki* values generated from the FA competition assay can then be used to rank peptide or small-molecule candidates that specifically bind to and block the toxin’s P-stalk sites. This strategy has been successfully employed by our group to rank the binding affinity of new P-stalk inhibitor candidates of RTA and further improve affinity through structure-guided drug design. Early fragment-based discovery identified CC10501 as a specific P-stalk inhibitor with a *Ki* of 32 µM [60]. Use of the FA assay described above led to incremental improvement in binding affinity, from RU-NT-93 (*Ki* = 3 µM), to RU-NT-192 (*Ki* = 1 µM), and finally to RU-NT-206 (*Ki* = 0.6 µM) [59,61,62]. RU-NT-206 has an approximately 50-fold more potent binding affinity compared to CC10501 and is the most P-stalk potent binding inhibitor described to date [61]. The rapid screening approach has proven indispensable in the rapid identification of hit compounds and significantly reduced dependency on costly and time-consuming functional assays for inhibitor screening. Overall, the FA competition approach enables rapid, cost-effective screening of multiple candidate inhibitors simultaneously, making it a critical tool for RIP-P-stalk binding inhibitor discovery and optimization studies.

## 4. FRET-Based Assays for Large-Scale Compound Screening

While the FA competition assay is a robust method for screening compounds that specifically bind to and block RIP P-stalk binding sites, there are limitations that could interfere with readouts. The FA assay relies on raw fluorescence measurements from the sample to calculate anisotropy values. This makes anisotropy values susceptible to background fluorescence from both protein and inhibitor candidates, increasing noise and potentially limiting the pool of small-molecule scaffolds that can be explored [93]. The noise reduces the dynamic range of inhibitor studies and increases assay-to-assay scatter, which can interfere with inhibitor SAR optimization [93]. In contrast to FA, TR-FRET utilizes specialized lanthanide fluorophores with long-lasting fluorescence, which allows background fluorescence from buffers, proteins, or compounds to be quenched prior to readout [94,95]. TR-FRET can be adapted for high-throughput inhibitor screening using the same P11 probe described in the FA assay (Section 3) [94,95]. A TR-FRET competition assay can also be adapted for initial primary screens of large compound libraries, such as the Maybridge collection, due to its robust, high-throughput nature and its tolerance for potential compound interference [93,96]. Importantly, these initial primary screens using a TR-FRET competition assay would measure specific binding rather than total binding readouts observed with SPR. This enhances the P-stalk binding inhibitor workflow and reduces the number of expensive confirmation assays (like X-ray crystallography) of binding hits that need to be conducted during initial compound library screens.

### 4.1. Principles of TR-FRET for HTS Campaigns

FRET is a distance-dependent physical process in which energy is transferred non-radiatively from an excited donor fluorophore to a nearby acceptor fluorophore (typically <10 nm apart) [95]. In a binding assay, the donor is attached to one binding partner and the acceptor to the other. When they associate, FRET occurs, generating a signal at the acceptor’s emission wavelength. TR-FRET enhances this principle for HTS by using a lanthanide chelate (e.g., Terbium or Europium [Eu]) as the donor [94,95]. Lanthanides have two key properties: a large Stokes shift and a long fluorescence lifetime (microseconds to milliseconds), which is orders of magnitude longer than the background fluorescence from library compounds, plastics, or biological media (nanoseconds) [94,95]. By introducing a time delay (a “time gate”) between the excitation pulse and signal detection, this short-lived background fluorescence is allowed to decay completely. The only signal measured is the long-lived emission from the FRET interaction, resulting in an exceptionally high signal-to-noise ratio and a high Z’ factor, both of which are critical for the reliability of HTS campaign [94,97]. The enhanced sensitivity of a TR-FRET-based HTS assay potentially offers significant advantages over existing FA-based competition assays, especially for inhibitor candidates approaching sub-micromolar and nanomolar binding affinities.

### 4.2. Designing a Competitive TR-FRET Assay for the Toxin P-Stalk Pocket

A practical and robust TR-FRET assay for screening inhibitors of the toxin–ribosome interaction can be designed in a competitive format analogous to the FA assay.

1. Reagent Preparation: The target protein (e.g., RTA or Stx2a) can be expressed with a C-terminal polyhistidine (His) tag, enabling an indirect, oriented labeling strategy through commercially available anti-His antibody or nanobody conjugated to a lanthanide donor (e.g., Tb-anti-His) [97]. This indirect labeling strategy eliminates the need for direct chemical modification of the toxin, which could potentially alter its conformation or activity. The high-affinity P11 peptide probe is chemically synthesized and labeled at the N-terminus with a compatible acceptor fluorophore such as BODIPY-TMR-X [97].
2. Assay Principle: In the assay well, the Tb-labeled anti-His antibody binds to the His-tagged RTA or Stx2A1, creating the donor complex. Subsequent binding of the acceptor-labeled P11 peptide to the P-stalk binding pocket of RTA positions the Tb donor and acceptor fluorophores in close proximity to produce efficient FRET and generate a high TR-FRET signal (a ratio of acceptor emission to donor emission) [93]. The acceptor-labeled P11 peptide then binds to the P-stalk pocket of RTA. This brings the Tb donor and the acceptor fluorophore into close proximity, enabling efficient FRET and generating a high TR-FRET signal (a ratio of acceptor emission to donor emission) [93].
3. Inhibitor Action: The assay is performed in the presence of compounds from a screening library. If a compound binds to the P-stalk pocket of RTA, it will competitively displace the acceptor-labeled P11 probe. This separation of the donor and acceptor disrupts the FRET process, leading to a concentration-dependent decrease in the TR-FRET signal [93,97]. This decrease is the primary readout used to identify “hit” compounds.

FA and TR-FRET assays are complementary approaches for identifying and ranking inhibitors that disrupt RIP-ribosome interactions. FA directly measures changes in rotational diffusion of a labeled probe upon binding, enabling quantitative determination of binding affinities and reliable ranking of inhibitors that displace the probe from the ribosome-binding site, often with lower susceptibility to signal amplification artifacts. In contrast, TR-FRET is highly amenable to HTS, providing a sensitive and robust platform for primary hit identification based on energy transfer between donor and acceptor fluorophores when the interaction is intact. However, TR-FRET signals can be influenced by compound interference, aggregation, or optical artifacts, necessitating secondary validation. As a result, TR-FRET is typically well suited for rapid hit discovery, whereas FA offers greater precision for confirming and ranking inhibitors based on their ability to disrupt toxin–ribosome interactions.

## 5. Orthogonal Functional Assays for Hit Validation and Mechanistic Elucidation

A compound is not considered a validated “hit” until it has been shown to inhibit the toxin’s biological activity in a functional context. For example, compounds with highly rigid, planar, aromatic structures often have intrinsic fluorescence, contributing to false-positive hits in the FA and TR-FRET assays. Additionally, SPR readouts are susceptible to non-specific binding, leading to the apparent detection of tightly binding compounds that have minimal or no effect on toxin depurination activity. Without the in vitro functional assays, any cellular result would be hard to interpret. A compound could fail in cells because it is not active on the toxin, or because of unrelated issues like poor permeability, instability, or efflux. There would be no way to distinguish these cases. Orthogonal assays, such as those that measure a downstream biological effect, are therefore essential to confirm the mechanism of action and eliminate false positives arising from assay artifacts. In vitro depurination assays provide that missing link by showing whether binding actually inhibits depurination.

### 5.1. Eukaryotic Ribosome Depurination Assay: On-Target Validation

**Figure 4:**
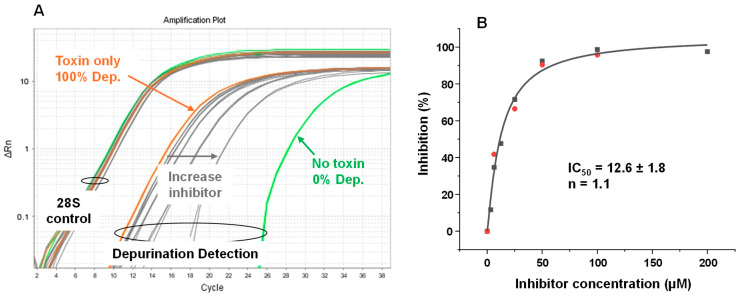
In vitro qRT-PCR assay to determine the *IC*_50_ of toxin-mediated depurination of rat ribosomes by small molecule inhibitors. (**A**). qRT-PCR amplification plot from inhibition assay. RNA prepared from rat liver ribosomes treated with toxin (orange), toxin plus inhibitor (gray) or untreated (green) were used in qRT-PCR with primers specific for total 28S rRNA or depurinated rRNA. Samples with no rRNA depurination (green) require more PCR cycles for detection compared to samples with high rRNA depurination (orange). Inhibition of depurination (gray) shifts amplification to the later cycles indicating decreased rRNA depurination. Use of total 28S rRNA serves as an RNA input control for each experimental condition. The percentage of inhibition was calculated using toxin only as 100% depurination. (**B**). the percentage of inhibition at different inhibitor concentrations was fit using Hill function to determine the *IC*_50_ of an inhibitor.

The ribosome depurination assay provides a direct, quantitative measure of the toxin’s primary catalytic activity on eukaryotic ribosomes, serving as the ultimate confirmation of on-target engagement [43,58,62,63]. The principle relies on a unique property of the reverse transcriptase enzyme when it encounters the abasic site on the 28S rRNA created by the toxin. The enzyme either stalls or, in many cases, incorporates a base opposite the gap [58,98,99,100]. This sequence alteration in the resulting complementary DNA (cDNA) can be detected with high specificity using quantitative real-time PCR (qRT-PCR). A primer is designed whose 3’ end corresponds to the site of depurination; specific amplification occurs only from cDNA templates derived from depurinated rRNA. By comparing the amount of this product to the amount of total 28S rRNA (measured with a separate primer set) (Figure 4A), a precise and quantitative measure of the extent of depurination can be obtained (Figure 4B) [58,61,62,101].

The in vitro protocol against rat ribosomes, as successfully employed in the validation of novel RTA inhibitors, involves a clear sequence of steps [43,58,62]:

1. Ribosome treatment: Samples containing rat ribosomes are prepared and treated with toxin for 5 min (e.g., 0.2 nM RTA or Stx2a1) in the presence of a serial dilution of the inhibitor compound. Controls with no compound (positive control) and no toxin or compound treatment (negative control) will also be conducted.
2. RNA extraction and cDNA synthesis: After the 5-min incubation, the RNA is extracted from the ribosomes. The RNA is then reverse-transcribed to generate cDNA for subsequent qPCR.
3. Quantitative PCR (qPCR): Two separate qPCR reactions are performed for each sample. The first reaction uses a primer set specifically designed to amplify cDNA derived from depurinated 28S rRNA using the sequence-specific primer for the abasic site base change. The second reaction uses a primer set that amplifies a region of the 28S rRNA unaffected by the toxin, serving as an internal control to quantify the total amount of 28S rRNA in the sample.
4. Data analysis: Cycle threshold values derived from the qPCR assay are analyzed using the comparative Ct (^ΔΔ^Ct) method. The amount of depurinated rRNA is normalized to the amount of total 28S rRNA for each sample, and then the normalized abundance of depurinated rRNA in experimental samples is compared to the normalized amount from a control sample. The percent inhibition of depurination is then calculated for each inhibitor concentration relative to the toxin-only control. These data are plotted to generate a dose–response curve, from which the half-maximal inhibitory concentration (*IC*_50_) is determined. The *IC*_50_ represents the concentration of the compound required to inhibit 50% of the toxin’s depurination activity of purified rat ribosomes.

A compound with a potent *IC*_50_ in the in vitro depurination assay has been shown to protect purified ribosomes from the toxin. A subsequent mammalian depurination assay utilizes live mammalian cells (e.g., Vero cells) to demonstrate an inhibitor’s efficacy in a living cell, where it must overcome additional hurdles such as membrane permeability, metabolic stability, and potential efflux [43,58,62]. The mammalian cell depurination assay utilizes a similar principle to the in vitro depurination assay against rat ribosomes, except that treatment and RNA extraction are done in the context of cells:

1. Cell treatment: Adherent mammalian cells (e.g., Vero cells, which are highly sensitive to these toxins) are cultured in multi-well plates. The cells are then treated with a fixed, sub-lethal concentration of the holotoxin (e.g., 200 pM ricin or 2 nM Stx2a) in the presence of a serial dilution of the inhibitor compound. Control wells include cells with no treatment, cells with toxin only (0% inhibition), and cells with compound only.
2. RNA extraction and cDNA synthesis: After a defined incubation period (e.g., 2 h), the cells are lysed, and total RNA is extracted. The RNA is then reverse-transcribed to generate cDNA for subsequent qPCR.

The remaining depurination steps are identical to the in vitro assay described above. The functional readout of the mammalian depurination assay is the half-maximal effective concentration (*EC*_50_). The *EC*_50_ represents the concentration of the compound required to inhibit 50% of the toxin’s depurination activity inside the cell. A compound that demonstrates a potent *EC*_50_ in the cell-based assay has successfully passed a series of critical, implicit tests. It has proven to be:

- Cell-permeable: It can cross the plasma membrane to reach the cytosol.
- Metabolically Stable: It is not immediately degraded or inactivated by cellular enzymes.
- Not Subject to Efflux: It is not rapidly pumped out of the cell by efflux pumps.
- Functionally Active: It successfully engages the toxin at its intracellular site of action and inhibits its primary catalytic function.

A strong correlation between a compound’s binding affinity (*Ki*) from biophysical assays and in vitro (*IC*_50_) and cellular efficacy (*EC*_50_) from the depurination assays provides the most compelling evidence that the compound is a high-quality lead operating through the intended mechanism of action [62]. This result provides high confidence to advance the compound into more complex studies, such as cytotoxicity assays and, ultimately, in vivo animal models of toxicity.

### 5.2. Cell-Free and Cell-Based Translation Assays: Measuring the Functional Consequence

A crucial functional validation step is to determine whether a compound that binds to the toxin can prevent it from inhibiting protein synthesis. Cell-free translation assays provide a direct measure of this functional outcome [58,102]. Cell-free translation assays contain all the necessary components for protein synthesis (ribosomes, tRNAs, amino acids, energy sources, and initiation/elongation factors) and are combined in an Eppendorf tube. An mRNA template, typically encoding a reporter protein such as luciferase, is added, and the rate of protein synthesis is measured. When a toxin like RTA or Stx2A1 is added, translation is inhibited. A successful inhibitor will rescue this inhibition, restoring protein synthesis [52].

For many years, the standard for cell-free translation has been commercially available kits based on lysates from rabbit reticulocytes (RRL), yeast, or wheat germ [102,103,104,105]. While convenient, these systems have significant limitations. They are derived from non-human sources, which raises the possibility of species-specific differences in the translation machinery that could affect inhibitor efficacy. Furthermore, they are “complete” lysates, meaning all cellular components are mixed, which prevents a detailed mechanistic dissection of an inhibitor’s action. However, it is impossible to know if the inhibitor is acting on the ribosome, the toxin, or some other translation factor in the lysate [102].

A more advanced and powerful approach is the use of a human cell-derived tripartite system [102]. This system involves the fractionation of human cell lysate into three distinct, functionally competent components: (1) purified, translation-competent ribosomes; (2) a ribosome-depleted cytoplasmic extract containing all necessary soluble factors (tRNAs, synthetases, initiation/elongation factors); and (3) an mRNA template pool. This system offers several key advantages for inhibitor validation:

1. Human relevance: By using components derived entirely from human cells, it eliminates concerns about species-specific artifacts and provides a more accurate assessment of an inhibitor’s potential in a human context.
2. Mechanistic flexibility: The fractionated nature of the system allows for powerful mechanistic experiments. For example, to confirm an inhibitor acts directly on the toxin–ribosome interaction, one can pre-incubate the toxin and ribosomes with the inhibitor before adding the cytoplasmic extract and mRNA. This level of control is impossible in a complete lysate system.
3. HTS compatibility: The prepared human lysates and ribosome fractions exhibit pronounced thermostability at room temperature (20−25 °C), a critical feature that makes them compatible with the robotic liquid handling systems used in automated, higher-throughput secondary screening.

The readout for cell-free translation assays can be tailored to the experimental need. For higher-throughput validation, the synthesis of a luciferase reporter, measured via luminescence, is a rapid and sensitive method [58,102]. However, this approach is vulnerable to a significant pitfall: direct interference of library compounds with the reporter enzyme itself [52]. It has been shown that small molecules, particularly those structurally similar to luciferin, can directly interact with firefly luciferase, either inhibiting or, paradoxically, enhancing its activity, leading to false-positive or false-negative results unrelated to translation [52]. To mitigate this risk, a mandatory counter-screening strategy must be implemented. Any hit identified using a luciferase-based readout must be retested using an orthogonal, non-reporter-based method. Two such robust methods are:

1. Radiolabel incorporation: The incorporation of a radiolabeled amino acid, such as ^35^S-methionine, into newly synthesized proteins provides a global and unbiased measure of total protein synthesis. The resulting proteins can be visualized by autoradiography after gel electrophoresis or measured via total protein isolation using a manifold system/glass filter/scintillation counter [106].
2. Biotinylated tRNA incorporation: A non-radioactive alternative involves using a specialized tRNA, such as Transcend tRNA, which is pre-charged with a biotin-labeled lysine. Nascent proteins incorporating this tag can be detected by Western blot using streptavidin-horse radish peroxidase or quantified by mass spectrometry after streptavidin pulldown [107].

Furthermore, a critical control experiment involves running the assay with the test compound but without the toxin. This will immediately flag compounds that are general translation inhibitors or that interfere with the reporter system, allowing them to be eliminated from further consideration. By systematically incorporating these counter-screens, potential artifacts are proactively identified and removed, ensuring that only true, on-target inhibitors of the toxin’s action advance in the screening cascade.

Cell-free translation assays, therefore, represent a critical bridge between biophysical binding assays and cellular validation, providing a direct functional readout of whether candidate compounds can rescue protein synthesis from RIP-mediated inhibition. While traditional lysate-based systems offer convenience and scalability, their inherent limitations in physiological relevance and mechanistic resolution can obscure the mode of action of candidate inhibitors. In contrast, advanced fractionated human systems provide a more physiologically relevant and experimentally flexible platform, enabling precise dissection of inhibitor mechanisms, particularly those targeting toxin–ribosome interactions. However, because cell-free systems do not fully capture the complexity of living cells, cell-based translation assays constitute an essential complementary validation step [43,55,70,108]. By measuring protein synthesis in intact cells exposed to RIPs, these assays assess whether candidate inhibitors retain activity in the presence of cellular uptake mechanisms, intracellular trafficking pathways, metabolic processes, and potential off-target effects. Cell-based approaches also provide an early indication of compound permeability, stability, and cytotoxicity, thereby helping to distinguish inhibitors that are effective under physiologically relevant conditions from those whose activity is restricted to simplified in vitro systems. Importantly, integrating orthogonal readouts and rigorous counter-screening strategies across both cell-free and cell-based platforms is essential to eliminate assay artifacts and ensure accurate interpretation of results. Collectively, these approaches establish a robust framework for functional validation, allowing the prioritization of inhibitors that genuinely restore translation and act through the intended mechanism, thereby strengthening downstream therapeutic development efforts.

### 5.3. Cellular Cytotoxicity Assay: The Final Consequence

While the depurination and cell-free translation assays confirm functional inhibition of toxin depurination and protein synthesis, respectively, a broader assessment of a compound’s ability to protect living cells from toxin-induced death is a critical next step. The CellTiter-Glo Luminescent Cell Viability Assay is a robust method for this purpose [61,109]. The assay quantifies ATP, an indicator of metabolically active, viable cells. The amount of ATP is directly proportional to the number of living cells in culture. The assay utilizes a thermostable luciferase that, in the presence of ATP, generates a stable “glow-type” luminescent signal with a half-life of several hours. This simple “add-mix-measure” format, where a single reagent is added directly to the cells in their culture medium without any washing or extraction steps, makes it exceptionally well-suited for automated HTS in 384-well or even 1536-well formats.

To set up the assay, a cell line sensitive to the toxins, such as Vero cells, is seeded in multi-well plates. The cells are then treated with compounds at different concentrations, followed by a lethal dose of the holotoxin (e.g., ricin or Stx). The cell with compound only and the cells with toxin only are set on the same plate as negative and positive controls. After a prolonged incubation period (e.g., 48 h) to allow for cytotoxicity to manifest, the CellTiter-Glo reagent is added. The resulting luminescence is measured, where a high signal indicates a large population of viable cells and thus a protective effect of the compound [61]. This assay serves as a final consequence of protection to evaluate the inhibitor that not only binds the target and inhibits its function in vitro but also translates that activity into a tangible protective effect in a cellular context.

The other function of this assay is to evaluate the cytotoxicity of the compounds themselves. Using the same set as above, the differences are (1) not including toxins and (2) increasing the compound concentration to 10 to 100 times their protection dose. The idea is that compounds that do not exhibit cellular toxicity at more than 100 times their protective dose are generally considered not toxic to mammalian cells.

The downside of this assay is that, because it relies on luminescent detection, some compounds can interfere with the signal readout. The interference could be either an increase or a decrease in reading. In some cases, if the interference is minor, the results can be adjusted using the control reading from the compound alone. However, if the interference exceeds 20%, it is very difficult to adjust, especially when compounds decrease the signal. In this case, the assay cannot determine whether the compound induces cell death or interferes with the assay readout. Thus, a candidate compound that interferes with the readout cannot be used to conduct either the compound toxicity assay or the protection assay. Other methods need to be developed to overcome this limitation.

## 6. Tiered Assay Strategy for Toxin P-Stalk Inhibitor Development

To enhance the workflow for RIP-P-stalk inhibitor development, we employed a multi-tier strategy that breaks inhibitor development into distinct stages, progressing from primary HTS against purified toxin to mammalian cellular assays. These steps are summarized in Table 1. The first stage uses HTS methods, including SPR and TR-FRET, to identify initial hit compounds from fragment libraries that bind the toxin’s P-stalk pocket. The specific binding of these hit compounds is confirmed by the FA competition assays, and rank-order inhibitor affinity (Ki) comparisons are used for structure-activity relationship (SAR) and subsequent chemical optimization. For inhibitors with strong binding (*Ki* < 10 µM), in vitro functional validation assays are then conducted to confirm that the inhibitors protect eukaryotic ribosomes from depurination. Compounds that protect against in vitro toxin activity are then tested in mammalian cells, which confirms whether the inhibitors can penetrate cells and protect ribosomes in the context of a complex cellular environment. The use of a tiered approach greatly speeds up the initial characterization of inhibitors and frontloads HTS assays, which are far more cost-effective and less time-consuming than later-stage cell-based assays.

The tiered screening cascade provides a structured and efficient framework for the discovery and optimization of inhibitors targeting RIP–P-stalk interactions. By progressively filtering compounds from high-throughput biophysical binding assays through mechanistic in vitro functional validation to mammalian cell-based confirmation, the workflow ensures only molecules with verified binding, functional activity, and cellular efficacy advance through the pipeline. Early prioritization of SPR and TR-FRET-based screening maximizes throughput and cost efficiency, while downstream FA, cell-free translation, and depurination assays add increasingly stringent layers of validation, reducing false positives and clarifying the mechanism of action. Importantly, this staged approach integrates affinity, function, and cellular relevance into a unified decision-making strategy, thereby accelerating SAR development and improving the likelihood of identifying robust, cell-active lead compounds suitable for preclinical advancement.

Each tier of the inhibitor discovery cascade employs distinct assay formats, each offering specific methodological strengths while also introducing inherent limitations that must be carefully considered during data interpretation. To ensure a balanced and transparent evaluation framework, Table 2 summarizes the key advantages and disadvantages associated with each assay used throughout the RIP–P-stalk inhibitor development pipeline. Together, these complementary methods enable a progression from rapid, high-throughput binding detection to increasingly stringent functional and cellular validation, while highlighting the trade-offs between throughput, physiological relevance, mechanistic resolution, and experimental complexity at each stage of the workflow.

We have successfully integrated this tiered assay strategy to develop inhibitors targeting the P-stalk binding site of RTA. Initial fragment screens with SPR identified the small fragment CC10501 as a specific RTA P-stalk binding inhibitor with favorable binding orientation and potential for optimization [60]. We then employed the tiered workflow, starting with FA competition screens for binding potency, then moving to in vitro depurination assays with rat ribosomes, and finally testing the best candidates in Vero cells. This approach allowed us to structurally optimize CC10501 and identify potent (low micromolar) inhibitors of RTA-ribosome binding [59,61,62]. Importantly, enhanced inhibitor binding potency to RTA was directionally linked to improved in vitro and mammalian cell depurination protection [59,61,62]. The most potent inhibitor described by our group to date, RU-NT-206, improved binding potency by approximately 50-fold, from a *Ki* of 32 µM with CC10501 to 0.6 µM with RU-NT-206 [61,62]. Additionally, the in vitro depurination *IC*_50_ improved from 427 µM with CC10501 to 18 µM with RU-NT-206, a 24-fold improvement [61,62]. Critically, the *EC*_50_ of RU-NT-206 was 29 µM, whereas CC10501 had no determinable *EC*_50_ due to poor cellular activity [61,62]. However, we found that RU-NT-206 was structurally incompatible with the cell toxicity assay and results were indeterminable, highlighting one of the major drawbacks indicated in Table 2 [61].

Overall, integrating a tiered assay approach streamlines the inhibitor discovery workflow by combining the strengths of complementary techniques while mitigating their individual limitations. This staged strategy improves efficiency by enabling rapid early-stage screening, followed by progressively more stringent validation, thereby reducing time and resource expenditure associated with late-stage failures. By systematically filtering compounds from initial binding detection to functional and cellular validation, the pipeline enhances confidence in hit-selection and prioritization.

## 7. Discussion

RIPs, such as ricin and Shiga toxin, represent a uniquely challenging class of toxic enzymes due to their high catalytic efficiency, intracellular mode of action, and ability to irreversibly damage ribosomes by depurinating the sarcin–ricin loop. Despite decades of research, the absence of approved therapeutics underscores both the biological complexity of these toxins and the limitations of existing drug discovery strategies. This review highlights how systematic integration of biophysical, biochemical, and cellular assays can help overcome current barriers and accelerate the identification of effective inhibitors. A key theme emerging from the evaluated methodologies is the importance of targeting toxin–ribosome interactions rather than the catalytic active site alone. The spatial separation between ribosome binding interfaces and catalytic centers in many RIPs provides an opportunity for allosteric inhibition. A tiered assay strategy was employed to balance throughput, specificity, and physiological relevance in the identification and validation of inhibitors targeting the P-stalk binding site.

Active-site inhibitors against RIPs have struggled largely because the catalytic pocket is unusually large, shallow, and highly polar, evolved to accommodate an RNA substrate rather than a small drug-like molecule. Thus, it is difficult to achieve high affinity and specificity with conventional small molecules. Most bind weakly or are outcompeted by the natural ribosomal substrate. By contrast, inhibitors that target the ribosome-binding interface (such as the P-stalk interaction site) tend to work better for a few key reasons. First, they exploit a functionally essential protein–protein interaction. Both toxins rely on binding to the ribosomal P-stalk to correctly position themselves for catalysis. Blocking this interaction effectively prevents the toxin from ever engaging its substrate, so inhibition occurs upstream of catalysis rather than competing within the active site itself. Second, the ribosome-binding site is often more structurally defined and druggable than the catalytic pocket. It includes pockets and grooves shaped for peptide interactions (e.g., the conserved C-terminal tails of P-proteins), which are more amenable to mimicry by peptides or small molecules with higher affinity and specificity. Third, these inhibitors benefit from an avidity and localization effect. In cells, ribosome binding dramatically increases the local concentration of toxin at its substrate. Disrupting this interaction reduces effective concentration and catalytic efficiency, even if the active site remains intact.

The allosteric inhibition arises because ribosome binding is not just for recruitment; it also induces or stabilizes a catalytically competent conformation of RTA [61]. When inhibitors block this interaction, they prevent proper orientation of the toxin relative to the sarcin-ricin loop of the large rRNA. They disrupt long-range conformational coupling between the ribosome-binding surface and the active site, leading to subtle but important changes in active-site geometry, dynamics, or electrostatics, reducing catalytic turnover. In other words, even though the inhibitor does not occupy the catalytic pocket, it decouples binding from catalysis, shifting the enzyme into a less active or inactive state. This is a classic case of allosteric control, where interference at one site (the ribosome interface) functionally disables another (the active site). Overall, targeting the ribosome interaction site is more effective because it blocks a prerequisite step for toxicity and leverages structural features more compatible with high-affinity inhibitor design, while simultaneously exerting indirect (allosteric) suppression of catalytic activity.

To enhance the workflow of developing inhibitors that block RIP-ribosome interactions, we have integrated a tiered approach for hit identification and optimization. The primary assay for identifying hits that inhibit ribosome interactions of RIPs utilizes SPR to screen fragment libraries. SPR offers label-free detection and provides real-time kinetic information, enabling the characterization of association and dissociation behavior. Subsequent hit confirmation and SAR development were performed using the FA competition assay, which proved particularly effective for quantifying site-specific interactions and mitigating non-specific interactions observed with SPR. A TR-FRET-based competition assay can also be integrated to expand the assay dynamic range, enabling the measurement of inhibitor binding affinities in the sub-micromolar and nanomolar range, and can be integrated at the primary assay level to complement SPR and quantify specific binding affinities of fragment library hits. Finally, cellular validation was achieved using both an in vitro and mammalian cell depurination assay quantified by qRT-PCR, which confirms inhibition of toxin catalytic activity in a physiological context. This assay represents a stringent test of compound efficacy, as it integrates multiple parameters, including cell permeability and intracellular stability. The mammalian cell depurination assay can be complemented by a cellular toxicity assay that assesses an inhibitor candidate’s ability to prevent RIP-induced cell death and can also be adapted to directly measure compound toxicity in mammalian cells. However, significant technical issues with this method exist, and care should be taken before workflow integration.

Taken together, this integrated workflow leverages the strengths of each assay while compensating for their individual limitations. Early-stage assays prioritize throughput but are prone to artifacts, whereas later-stage assays offer greater specificity and biological relevance. Notably, the FA assay emerged as a critical method for accurately assessing site-specific binding, bridging the gap between initial screening and functional validation. This tiered approach ensures that compounds advancing through the pipeline are not only high-affinity binders but also functionally active and effective in a cellular context.

### Gaps and Future Prospects

Despite significant advances in understanding the structure and mechanism of RIPs, the development of effective small-molecule inhibitors remains challenging. Current studies frequently treat binding assays, depurination assays, and cell-based validation platforms as independent methodologies, resulting in a fragmented approach to inhibitor discovery. Consequently, there remains a need for integrated screening workflows that facilitate the progression of candidate molecules from initial target engagement studies through functional validation in biologically relevant systems. This review proposes such a workflow by linking complementary assay platforms for the systematic identification and validation of small-molecule inhibitors of RIPs. Nevertheless, several important challenges remain.

A major limitation of existing screening strategies is their reliance on simplified experimental systems that do not fully recapitulate the complexity of toxin uptake, intracellular trafficking, ribosome engagement, and host-cell responses. As a result, compounds demonstrating activity in biochemical assays may not necessarily retain efficacy in cellular or in vivo settings. The off-target toxicity of compounds against cells can also interfere with the functional readout of cellular assays, including the mammalian depurination assay and cellular toxicity assay. Furthermore, the lack of assay standardization across laboratories complicates the comparison of inhibitor potency, selectivity, and mechanisms of action, thereby hindering the prioritization of lead compounds.

Future efforts should emphasize the incorporation of orthogonal validation approaches, improved assay reproducibility, and the development of standardized benchmarking criteria. The integration of structural biology techniques, including cryo-electron microscopy, X-ray crystallography, and nuclear magnetic resonance spectroscopy, will provide detailed mechanistic insights into toxin–inhibitor interactions and support structure-guided optimization. Such approaches may also facilitate the identification of novel targetable sites beyond the canonical ribosome-binding interface. Additionally, inhibitor efficacy in a single cell line (e.g., Vero cells) may not translate to other cell lines or animal models. This biological limitation emphasizes the importance of testing potent inhibitors in multiple cell lines and highlights the need to develop models that better recapitulate in vivo conditions, like organoid models.

Another important consideration is the structural and functional diversity of RIPs. Although these toxins share a conserved catalytic mechanism, variations in surface architecture, receptor interactions, intracellular trafficking pathways, and ribosome recognition may necessitate distinct inhibitor development strategies. The development of broad-spectrum inhibitors targeting conserved structural features remains an attractive objective but may prove challenging due to the need to balance efficacy with selectivity. Conversely, toxin-specific inhibitors may achieve greater potency and specificity but will require parallel optimization efforts for individual RIPs. Importantly, the assay platforms described in this review can be adapted and combined in different configurations to support structure-based inhibitor discovery against multiple toxin targets and alternative binding pockets.

Looking forward, advances in fragment-based drug discovery, HTS technologies, artificial intelligence-assisted drug design, and computational modeling are expected to play an increasingly important role in RIP inhibitor development. The integration of these emerging approaches with robust experimental screening and validation platforms has the potential to accelerate hit identification, lead optimization, and mechanistic characterization. Ultimately, improving the predictive relationship between in vitro screening outcomes and in vivo efficacy will be critical for translating promising inhibitors into clinically viable therapeutics against RIP-mediated diseases. The focus of this review is the biophysical and biochemical assays used for the identification and early validation of inhibitors to establish target engagement, mechanisms of action, and functional inhibition. Evaluation of pharmacokinetic properties, tissue distribution, and in vivo exposure will become relevant after compounds have demonstrated sufficient potency, selectivity, and cellular activity to justify progression into animal studies.

## Figures and Tables

**Table 1:** A multi-tiered assay cascade for toxin inhibitor discovery.

Tier	Stage	Assay	Purpose	Key Metric	“Go/No-Go” Criterion Example	References
1	Primary	SPR screen TR-FRET assay	To identify initial “hits” from a fragment screen that bind to the toxin, including the P-stalk pocket.	Binding affinity (*K_D_*, *Ki*)	The positive binders need to be tested by other methods to confirm the hit	[60,82,93,95,96]
2	Hit confirmation & SAR	FA competition assay	To confirm binding, accurately determine affinity (*Ki*), and establish structure-activity relationships (SAR).	*IC*_50_, *Ki*	Advance compounds with a confirmed *Ki* < 10 µM.	[76,79,85]
3	In vitro functional validation	In vitro depurination inhibition assay (qRT-PCR) Cell-free translation assay	To confirm that binding translates to functional inhibition of toxin activity.	*IC*_50_ (inhibition or translation)	Advance compounds that rescue translation with an *IC*_50_ < 25 µM and show no activity in counter-screens.	[42,52,102,109]
4	Cellular on-target validation	Mammalian cell depurination assay (qRT-PCR)	To confirm cell permeability and on-target inhibition of toxin catalytic activity in a physiological context.	*EC*_50_ (depurination)	A compound with a potent *EC*_50_ (e.g., <50 µM) is considered a validated lead for preclinical studies.	[32,44,63,100,101]
5	Cellular toxicity assay	Mammalian cell toxicity assay (different kits are available)	The final readout of compound protection	Toxin *IC*_50_ for cell death	Necessary step before a compound is considered for metabolic/stability and animal study	[61,109,110]

**Table 2:** The advantages and disadvantages of each assay in the toxin inhibitor study cascade.

Assay	Advantages	Disadvantages	References
SPR screen	Label-free detection, avoiding probe-related artifacts. Screen for weak binders, such as fragments. Provides real-time interaction data, including kinetic parameters (k_on_/k_off_). Can detect a broad range of affinities.	Requires immobilization of the target, which may alter binding behavior. Can detect non-specific or electrostatic interactions, especially at higher concentrations. Sensitive to experimental setup and surface chemistry.	[59,60,62,111]
TR-FRET competition assay	Enables high-throughput screening (HTS) of large compound libraries with strong scalability. Reduces background fluorescence contribution by buffer, compounds, and proteins. Wider dynamic range than the FA assay.	Requires fluorescent labeling of both protein and probe. Can still be susceptible to false positives (e.g., fluorescent compounds, quenchers, aggregators). Lanthanide fluorophores are significantly more expensive than traditional organic dyes.	[94,95]
FA competition assay	Measures binding in solution, preserving native interaction conditions. Provides accurate affinity determination (Ki), particularly for competitive binding. Effective for site-specific interaction analysis (e.g., P-stalk pocket targeting). Well-suited for structure–activity relationship (SAR) development. Reduces false positives seen with SPR.	Requires a carefully designed fluorescent probe. Susceptible to false positives (e.g., fluorescent compounds, quenchers, aggregators). Signal depends on probe displacement efficiency. Cannot provide kinetic information. Limited dynamic range for very weak or ultra-tight binders.	[59,61,79]
Depurination assays	Confirms on-target activity in a physiological context. Measures toxin catalytic inhibition directly on the ribosome. Incorporates cell permeability, stability, and intracellular engagement. Strong validation step for lead selection and preclinical relevance.	More complex and variable than in vitro binding assays. Lower throughput and more resource-intensive. qRT-PCR readout requires careful normalization and controls.	[32,58,71,106,110]
Cell-free and cell-based translation assays	Directly measures the functional inhibition of protein synthesis. Confirms that binding translates into biological activity. Eliminates compounds that bind but are mechanistically irrelevant. Provides a controlled system without cellular complexity.	Indirect measure of binding affinity. Cell-free assays may not fully reflect cellular conditions or permeability. Potential for off-target effects on translation machinery. Lower throughput than biochemical assays.	[52,102,112,113]
Cellular toxicity assay	Several commercial kits are available with plate-format reading that can get results quickly. Not only measure the protection of the compound against the toxin, but it can also test the cellular toxicity of compounds.	The mechanisms of toxin-induced cell death can result in different outcomes in the toxin-dependent readout. Compound interference is common, and kits are expensive.	[61,109,110,114]

## Data Availability

No new data were created or analyzed in this study.

## Abbreviations

The following abbreviations are used in this manuscript:

RIP	Ribosome-Inactivating Protein
RTA	Ricin Toxin A Chain
Stx	Shiga Toxin
Stx2A1	Shiga Toxin 2 A1 Subunit
P-stalk	Ribosomal P-Stalk
SPR	Surface Plasmon Resonance
PBS-P	Phosphate-Buffered Saline with Surfactant P20
DMSO	Dimethyl Sulfoxide
FA	Fluorescence Anisotropy
HTS	High-Throughput Screening
*F_b_*	Fraction Bound
*K_D_*	Equilibrium Dissociation Constant
*IC*_50_	Half-Maximal Inhibitory Concentration
*Ki*	Inhibitory Constant
qRT-PCR	Quantitative Reverse Transcription Polymerase Chain Reaction
PCR	Polymerase Chain Reaction
RNA	Ribonucleic Acid
rRNA	Ribosomal RNA
cDNA	Complementary DNA
FRET	Förster Resonance Energy Transfer
TR-FRET	Time-Resolved Förster Resonance Energy Transfer
BODIPY	Boron-Dipyrromethene
TMR	Tetramethylrhodamine
ATP	Adenosine Triphosphate
GTP	Guanosine Triphosphate
